# A survey of public eye-care behavior and myopia education

**DOI:** 10.3389/fpubh.2025.1518956

**Published:** 2025-02-18

**Authors:** Qianwen Liu, Moxin Chen, Tongtong Yan, Na Jiang, Qin Shu, Xiaoyi Liang, Zhuoran Tao, Xuefeng Yang, Wanqin Nie, Yonglin Guo, Xiaojing Li, Deyi Jasmine Zhu, Changjuan Zeng, Jin Li, Lin Li

**Affiliations:** ^1^Department of Ophthalmology, Shanghai Ninth People's Hospital, School of Medicine, Shanghai Jiao Tong University, Shanghai, China; ^2^Shanghai Key Laboratory of Orbital Diseases and Ocular Oncology, Shanghai, China; ^3^College of Arts and Science, New York University, New York, NY, United States

**Keywords:** myopia, science popularization, questionnaire survey, age, knowledge requirement

## Abstract

**Purpose:**

To investigate the prevalence of myopia, analyze public eye care behaviors, eye protective practices, and concerns regarding myopia among different age demographics. It also aimed to provide suggestions for improving the dissemination of science popularization about myopia, taking into account different age groups.

**Methods:**

Between May 2022 and July 2022, we gathered data from participants through online questionnaire surveys and subsequently analyzed the collected responses.

**Results:**

The research encompassed a total of 393 individuals, out of which 331 were identified as having myopia. The chi-square test revealed significant correlations between myopia and age (χ^2^ = 18.7, *P* = 0.002), comprehensiveness of eye examinations (χ^2^ = 5.0, *P* = 0.025), and adherence to the “20-20-20” rule (χ^2^ = 6.1, *P* = 0.014). Furthermore, there were notable variations in the frequency and comprehensiveness of eye examinations (χ^2^ = 14.0, *P* = 0.016), adherence to the “20-20-20” rule (χ^2^ = 25.267, *P* < 0.001), and levels of concern regarding myopia (χ^2^ = 63.8, *P* < 0.001) observed across different age groups. Participants aged 17 and below demonstrated a primary interest in acquiring fundamental knowledge about myopia. The 18–25 age group expressed a particular interest in strategies for managing myopia. Conversely, people between the ages of 36–45 are more concerned about whether the many rumors about myopia that have not been medically proven, such as “myopia can be cured,” are true.

**Conclusion:**

As the policies and awareness regarding myopia improve, younger people are adopting better eye-care behaviors in comparison to older populations. Enhancing visual health and reducing the occurrence of myopia requires the improvement of age-appropriate science popularization.

## 1 Introduction

The prevalence of myopia has significantly risen worldwide in the previous three decades. By 2050, it is projected that myopia will impact the lives of around 5 billion individuals (1). The National Health Commission of China conducted a survey in 2020, which revealed that 52.7% of children and adolescents in China suffer from myopia. Furthermore, the average age at which myopia begins has fallen by 3 years within the last 16 years (2). Uncorrected myopia has a greater likelihood of negatively affecting reading ability and academic performance in children and adolescents. In middle-aged and older individuals, myopia can lead to macular degeneration, retinal holes and tears, retinal detachment, and other serious complications that can result in irreversible visual impairment and even blindness (3–5).

Furthermore, myopia has a negative impact on psychological wellbeing and imposes a significant socioeconomic burden (6–9). Research has indicated that 25% of individuals with severe nearsightedness have symptoms of despair or anxiety (10). Annually, in the metropolitan regions of China, the direct medical expenses and indirect financial losses associated with myopia might potentially amount to a staggering 173.6 billion RMB (25.2 billion USD) (8), thereby adversely affecting society.

The prevention of myopia is crucial due to its potential to enhance people's visual and overall quality of life, while also alleviating the economic burden associated with this condition (11–13). Many studies have already proved the timely and effective eye-care behaviors can help people prevent myopia and delay myopia progression (14, 15). For example, conducting outdoor exercise regularly and following “one punch, one foot, one inch” (maintain a distance of 10 cm between the chest and the table, 33 cm between the eyes and the book, and 3.3 cm between the fingers and the tip of the pen while reading and writing) reduced the risk of myopia (16). However, a report from the World Health Organization (WHO) highlighted that interventions focused on health promotion in the field of eye care have received less attention compared to those aimed at secondary prevention or treatment. Therefore, in addition to establishing records of refractive development and standardizing the diagnosis and treatment of myopia, healthcare professionals should also engage in health education and promote knowledge about eye care. This is crucial in helping children and adolescents develop healthier habits of eye care, which play a vital role in preventing the onset of myopia and managing its progression (16). Over the past few years, there has been a gradual rise in the focus on visual health (17, 18). In order to enhance public knowledge about myopia and educate them on scientific approaches for preventing and managing myopia, medical professionals have produced numerous informative materials on the subject (19).

As social media develops, there are more and more science popularization works on health care, and researchers have found that these works could effectively improve the public's awareness of medical knowledge and the correctness of their behavior when confronted with relevant health issues (20, 21). Though there lacks study about the effectiveness on myopia, with the growing public awareness of myopia, the current abundance of science popularization materials on the subject fails to adequately cater to the specific needs of different age groups. Hence, it is imperative to expedite the advancement of popularizing myopia science. This study examined the distribution patterns of myopia in various age groups and the factors associated with it. In addition, the study examined problems linked to myopia in various age groups, along with their eye-care practices and ocular examinations. It also included recommendations for preventing and managing myopia, as well as improving the transmission of information about myopia to the public.

## 2 Materials and methods

### 2.1 Study design, setting, and participants

A cross-sectional study was undertaken between May 2022 and July 2022 to examine the state of refraction and eye-care practices across different age groups. Subjects were recruited by distributing questionnaires in hospital outpatient clinics or by disseminating them on the Internet. In our study, people with or without refractive errors, and willing to participate in the study were included. People unable or reject to answer the questionnaire were excluded. For children who weren't able to finish the questionnaire by themselves, their parents will finish it with integration of the child's reality. A total of 393 individuals completed the online questionnaire, all of whom met the criteria for validity by answering 90% or more of the questions.

### 2.2 Questionnaire design

The questionnaire (Supplementary material 1) was developed for this study, and administered via the internet platform “Wenjuanxing” using a process of simple random sampling. The Chinese version of the questionnaire is also publicly available on the website “Wenjuanxing” by the access https://www.wjx.cn/vm/wUgtX30.aspx. The questionnaire mainly had compulsory fields to guarantee the inclusion of all vital information and queries, which means that if subjects didn't choose an option of this question, they couldn't submit the questionnaire in the last. Survey results can be downloaded straight from the platform to prevent errors that may occur during manual data entry. The questionnaire has the following questions:

- Demographics: age and other relevant characteristics.
- Self-reported refractive status and perception of myopia: individuals' own accounts of their eye prescription status and their understanding of the connection between myopia and genetics, as well as any concerns they may have regarding myopia. In the study, “myopia” and other “refractive status” were defined as the refractive errors diagnosed through a vision examination in the hospital.
- Eye care habits and eye protective practices: If they maintain good eye protective practices, such as eye care habits and following the 20-20-20 rule, which involves taking a 20-s break and focusing on anything 20 feet away after every 20 min of continuous eye use, is important for maintaining eye health (22), the frequency of the examinations, and the thoroughness of the examinations.

The frequency of eye tests was categorized as regular or irregular. Regular eye examinations were defined as participants getting eye examinations at least once a year, while irregular eye examinations were regarded less frequent, occurring less than once a year. In order to ensure the thoroughness of eye examinations, comprehensive eye examinations included examinations of vision (uncorrected visual acuity and corrected visual acuity), astigmatism, axial axis, and fundus photography. On the other hand, less comprehensive eye examinations were defined as those in which participants underwent two or fewer examination items. The “Yes” option in the survey of compliance of “20-20-20” rule means that people have this habit, which is a subconscious behavior, while the “No” option means that they only do it when they think about it irregularly, or they never do it.

### 2.3 Statistical analysis

The obtained data were analyzed using the SPSS 21.0 program developed by IBM Corp. in Armonk, New York. The Chi-square test and Fisher-Freeman-Halton exact test were used to compare qualitative data between different groups. Afterwards, adjusted residuals (AR) were used to compare factors related to myopia among different age groups. A level of two-sided *P*-value < 0.05 or an absolute value of the AR greater than 2 was considered to be statistically significant.

## 3 Results

### 3.1 Demographic features of the study participants categorized by myopia status

**Figure 1:**
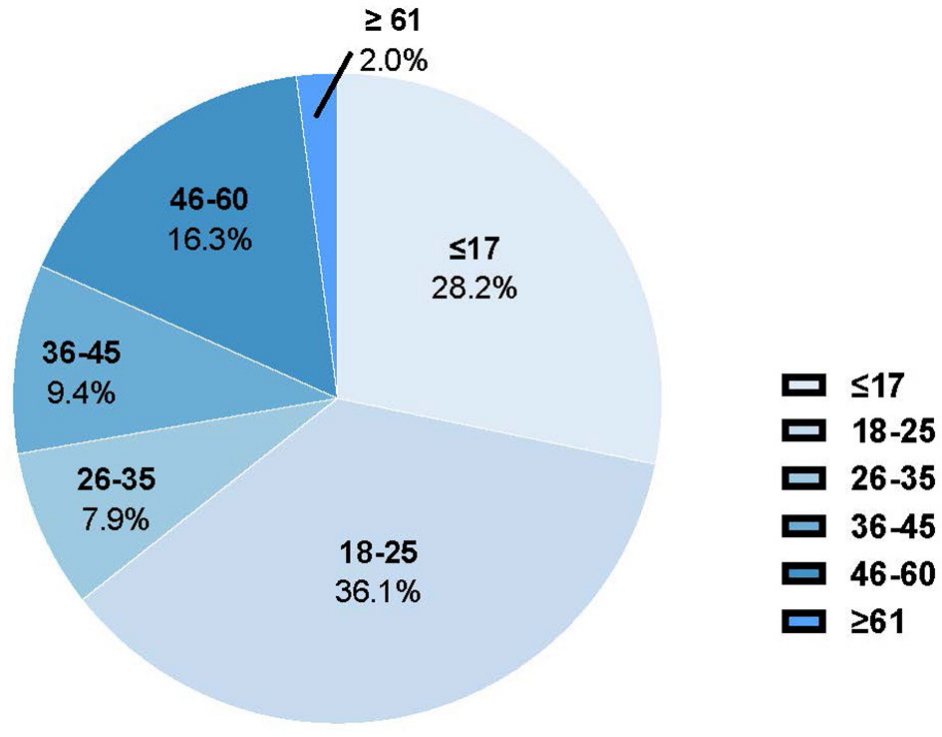
Age distribution of the study population.

**Figure 2:**
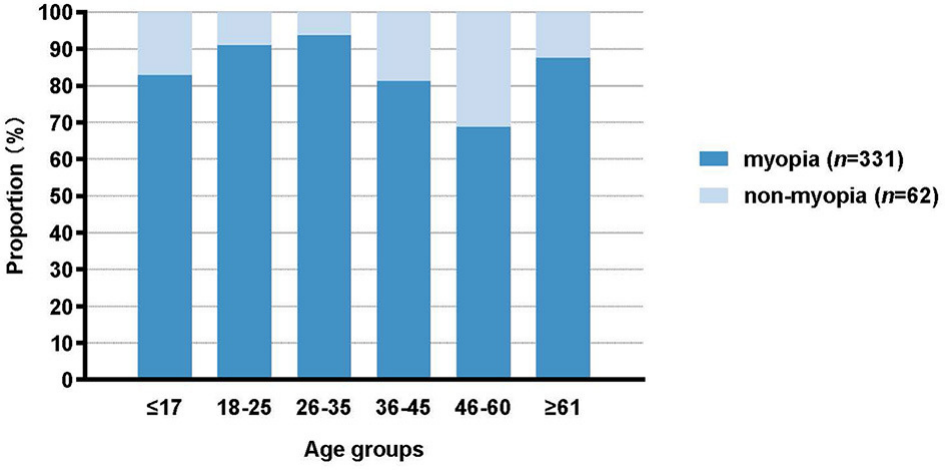
Myopia prevalence in different age groups.

Out of the 393 participants, 331 people (84.2%) were diagnosed with myopia, as indicated in Table 1. The participants had an median age range of 18–25 years. The age distribution of the participants is illustrated in Figure 1. There were 111 individuals (28.2%) who were 17 years old or younger, 142 individuals (36.1%) who were between 18 and 25 years old, 31 individuals (7.9%) who were between 26 and 35 years old, 37 individuals (9.4%) who were between 36 and 45 years old, 64 individuals (16.3%) who were between 46 and 60 years old, and 8 persons (2.0%) who were 60 years old or older. The prevalence of myopia varies among different age groups as shown in Figure 2. Among those aged 17 and below, the prevalence is 82.9%. For those aged 18–25, the prevalence is 90.9%. Among individuals aged 26–35, the prevalence is 93.6%. For those aged 36–45, the prevalence is 81.0%. Among individuals aged 46–60, the prevalence is 68.8%. Lastly, for those aged 60 and above, the prevalence is 87.5%. Participants in the age range of 18–25 showed a higher degree of myopia compared to other age groups, with an adjusted residual of 2.7.

**Table 1:** Characteristics of the study population by myopia status.^a^

	**Myopia**		***χ^2^* value**	***P*-value^b^**
	**Yes (*****n*** = **331)**	**No (*****n*** = **62)**		
**Age (years)**			18.7	0.002^*^
≤ 17	92 (82.9)	19 (17.1)		
18–25	129 (90.9)	13 (9.1)		
26–35	29 (93.6)	2 (6.4)		
36–45	30 (81.1)	7 (18.9)		
46–60	44 (68.8)	20 (31.2)		
≥60	7 (87.5)	1 (12.5)		
**Frequency of eye examination**			1.0	0.323
Regular	112 (86.8)	17 (13.2)		
Irregular	219 (83.0)	45 (17.0)		
**Comprehensiveness of eye**			5.0	0.025^*^
**examination**				
Comprehensive	200 (87.7)	28 (12.3)		
Less comprehensive	131 (79.4)	34 (20.6)		
**Compliance of the “20-20-20” rule**			4.8	0.028^*^
Yes	19 (67.9)	9 (32.1)		
No	312 (85.5)	53 (14.5)		

^a^Data values were number (percentage).

^b^*P* values for the overall difference were calculated using Chi-squared test, or Fisher-Freeman-Halton exact test, as appropriate. ^*^P < 0.05.

### 3.2 Study of factors related with myopia

A Chi-square test was performed on a survey of 393 individuals to investigate the impact of factors connected to myopia. The analysis showed significant statistical differences between the group with myopia and the group without myopia in terms of the prevalence of myopia based on age (χ^2^ = 18.7, *P* = 0.002), the thoroughness of eye examinations (χ^2^ = 5.0, *P* = 0.025), and adherence to the “20-20-20” rule (χ^2^ = 4.8, *P* = 0.028) (Table 1). Furthermore, while there was no significant statistical difference in the frequency of eye examinations between the group of people with myopia and the group without myopia (χ^2^ = 1.0, *P* = 0.323), a higher percentage of individuals in the myopic group (86.8%) underwent regular eye examinations compared to the non-myopic group (83.0%) (Table 1).

### 3.3 Comparison of factors related with myopia in different age groups

In order to conduct a more in-depth analysis of the distribution of factors connected to myopia in various age groups, we categorized the factors, such as the frequency and comprehensiveness of eye examinations, as well as compliance with the “20-20-20” rule, based on age groups. The results showed that there were substantial statistical differences in the distribution of eye examination frequency across different age groups (χ^2^ = 14.0, *P* = 0.016) (Table 2), the comprehensiveness of eye examinations (χ^2^ = 65.972, *P* < 0.001) (Table 3), and compliance with the “20-20-20” rule (*P* < 0.001) (Table 4). According to Table 2, those who were 17 years old or younger were more likely to have frequent eye tests, with an average rate of 3.5. Regarding the extent of eye examinations (Table 3), those who were 17 years old and younger were more likely to have thorough eye examinations (AR = 7.6), whereas those in the age groups of 18–25 (AR = 2.2), 26–35 (AR = 3.4), and 46–60 (AR = 2.8) tended to have less extensive eye tests. Concerning the adherence to the “20-20-20” eye care rule (Table 4), those who were 17 years old and younger were more likely to comply with this rule (AR = 4.8), whereas those who were between the ages of 18 and 25 (AR = 2.1) and 46 and 60 (AR = 2.4) often did not adhere to it. Nevertheless, the implementation of the “20-20-20” eye care guideline was comparatively low across all age demographics (Table 4).

**Table 2:** The frequency of eye examinations of participants in different age groups.^a^

	**Regular (*n* = 129)**	**Irregular (*n* = 264)**	***χ^2^* value**	***P*-value^b^**
**Age (years)**			14.0	0.016^*^
≤ 17	51 (3.5)	60 (−3.5)		
18–25	42 (−1.0)	100 (1.0)		
26–35	9 (−0.5)	22 (0.5)		
36–45	7 (−1.9)	30 (1.9)		
46–60	17 (−1.2)	47 (1.2)		
≥60	3 (0.3)	5 (−0.3)		

^a^Data values were number (AR).

^b^*P* values for the overall difference were calculated using Chi-squared test. ^*^P < 0.05. AR, adjusted residuals.

**Table 3:** The comprehensiveness of eye examinations of participants in different age groups.^a^

	**Comprehensive (*n* = 228)**	**Less comprehensive (*n* = 165)**	***χ^2^* value**	***P*-value^b^**
**Age (years)**			65.972	< 0.001^**^
≤ 17	98 (7.6)	13 (−7.6)		
18–25	72 (−2.2)	70 (2.2)		
26–35	9 (−3.4)	22 (3.4)		
36–45	20 (−0.5)	17 (0.5)		
46–60	27 (−2.8)	37 (2.8)		
≥60	2 (−1.9)	6 (1.9)		

^a^Data values were number (AR).

^b^*P* values for the overall difference were calculated using Chi-squared test. ^**^P < 0.001. AR, adjusted residuals.

**Table 4:** The compliance of “20-20-20” rule of participants in different age groups.^a^

	**Yes (*n* = 28)**	**No (*n* = 365)**	***P-*value^b^**
**Age (years)**			< 0.001^**^
≤ 17	19 (4.8)	92 (−4.8)	
18–25	5 (−2.1)	137 (2.1)	
26–35	0 (−1.6)	31 (1.6)	
36–45	3 (0.2)	34 (-0.2)	
46–60	0 (−2.4)	64 (2.4)	
≥60	1 (0.6)	7 (−0.6)	

^a^Data values were number (AR).

^b^*P* values for the overall difference were calculated using Fisher-Freeman-Halton exact test. ^**^P < 0.001. AR, adjusted residuals.

### 3.4 Comparison of concerns related to myopia in different age groups

In order to uncover the concerns of the participants regarding myopia in the context of science popularization, we proceeded to analyze the data based on different age groups. The results showed statistically significant differences (*P* < 0.001) (Table 5). Curiously, those who are 17 years old or younger show a higher level of interest with the causes of myopia (AR = 2.8) and if myopia can be reversed (AR = 2.7), with a particular focus on the fundamental scientific aspects of myopia. Conversely, individuals between the ages of 36 and 45 demonstrated more interest in rumors regarding myopia, such as the possibility of a complete cure or the potential worsening of myopia from wearing glasses. However, this age group no longer considers preventive measures for myopia, such as laser surgery, orthokeratology lenses, or low-concentration atropine eye drops, as a priority (AR = −2.9). Furthermore, while the variations in concerning of all five items (etiology, reversibility, rumors, management, others) among individuals aged 18–25 are not statistically significant, there is a noteworthy decline in interest regarding fundamental inquiries about the causes and potential reversibility of myopia, as compared to the age range of 0–17. Conversely, there is an increase in interest in the reality of some rumors as regard to myopia on social media and managing myopia (Table 5).

**Table 5:** Concerns related to myopia of participants in different age groups.^a^

	**Etiology**	**Reversibility**	**Rumors**	**Management**	**Others**	***P-*value^b^**
**Age (years)**						< 0.001^**^
≤ 17	28 (2.8)	62 (2.7)	51 (−1.7)	74 (−1.8)	3 (−1.6)	
18–25	19 (−1.4)	53 (−1.8)	89 (1.7)	120 (1.4)	4 (−1.9)	
26–35	4 (−0.3)	11 (−0.3)	11 (−1.3)	26 (1.5)	2 (0.4)	
36–45	2 (−0.7)	5 (−1.3)	22 (4.4)	6 (−2.9)	2 (1.0)	
46–60	5 (−1.2)	22 (0.2)	21 (−1.5)	42 (1.0)	7 (2.8)	
≥60	1 (−0.1)	2 (−0.3)	1 (−1.4)	5 (0.5)	2 (3.1)	

^a^Data values were number (AR).

^b^*P* values for the overall difference were calculated using Fisher-Freeman-Halton exact test. ^**^P < 0.001. AR, adjusted residuals.

## 4 Discussion

In recent decades, myopia has become a prominent public health concern in China. Prior research has indicated that the incidence rate of myopia in children and teenagers in China varies from 53.0% to 66.8%, however the prevalence of myopia among university students surpasses 90% (3, 23–26). The spatial distribution of myopia in China has already been shown (27–29), this study provided a detailed analysis of the myopia situation in different age groups. In our study, we found that the overall prevalence of myopia was 84.2%. Furthermore, we noticed a significant incidence of high myopia in all age groups. The prevalence of myopia among those aged 18–25 was specifically as high as 90.9%, which is similar to the earlier study conducted on this age group (30). This phenomenon may be related to factors such as the high intensity of work and study pressure, leading to extended periods of near-work time, as found in children and teenagers (16, 31, 32). Furthermore, a recent study indicated that decreased physical activity is associated with a greater incidence of myopia in college students (33). Concentrating on eye care habits in this study may have drawn more interest from persons with myopia, potentially leading to a higher total myopia rate.

The cause of myopia is complicated, encompassing both hereditary and environmental factors (34–38). Myopia is a condition that whose development cannot be reversed. Therefore, it is crucial to diagnose and treat it early through eye examinations (39, 40). This study investigated factors associated to myopia and discovered strong correlations between myopia and the thoroughness of eye examinations, adherence to the “20-20-20” eye care rule, and age. The prevalence of myopia was greater in individuals obtaining extensive eye examinations as opposed to those receiving less comprehensive eye examinations, suggesting an inclination among the general population to look for ophthalmic examinations as a method of managing myopia rather than avoiding it. Therefore, it presents the need that people, especially the parents and the children, should improve the awareness of control of the myopia, with regarding that timely and comprehensive eye examinations is a crucial part to prevent myopia in children. Besides, compared to the comprehensiveness of eye examinations, the frequency of eye examinations did not show a statistically significant difference between myopia and non-myopia groups. We suggest that this phenomenon may because there exist population who experience less comprehensive but regular examinations. Clearly, such simple examinations are often not sufficiently accurate, and due to their lack of rigor, they may not attract enough attention from those being examined. As a result, even regular eye examinations may not play a significant role in the prevention and control of myopia. In our data, such a population accounts for ~64.3% (82/129) of those undergoing regular eye examinations. Research has shown that adhering to the “20-20-20” eye care rule can successfully reduce visual fatigue and prevent the development of myopia (41, 42). Additionally, this study discovered that individuals who followed the “20-20-20” rule had a notably reduced rate of myopia. However, in contrast to a study that revealed more than 50% of pupils with grades ranging from 1 to 12 were unaware of the “20-20-20” eye care guideline (43), our investigation discovered that adherence to this rule was generally low among all age groups. Collectively, these findings demonstrate that there is a deficiency in knowledge regarding adequate eye care and the prevention of myopia. This highlights the necessity for widespread dissemination of complete information on myopia science in these areas.

Upon further comparison of factors related to myopia among different age groups, it was noted that persons aged 17 and below had a decreased prevalence of myopia compared to those aged 18–25. We also found that, overall, the 0–17 age group was more likely to undergo regular and comprehensive eye examinations compared to the 18–25 age group. In addition, 0–17 age group, demonstrated a greater propensity to follow the “20-20-20” eye care guideline, in comparison to other age groups. This suggests an increased understanding of eye health among children, adolescents, and their families. Nevertheless, additional endeavors by individuals, families, society, and government remain critical. Surprisingly, 18–25 age group compared to older groups showed likely and bad eye care habits. This may demonstrate a fact that in China, the awareness of eye care has not been developed over a long period of time, and for these young individuals who have missed the optimal period for controlling their vision, developing good eye care habits can still help prevent the progression of myopia (44).

Based on our research results and a high prevalence of myopia among all age groups, here we try to give some suggestions to different age group. Firstly, all individuals in this study demonstrated low adherence to the “20-20-20” eye care guideline. It is well known that modern people are highly dependent on electronic devices, and the “20-20-20” eye care guideline has been shown to be an effective means of reducing the symptoms of digital eye strain (DES), and has the advantage of being convenient and cost-effective compared to other strategies of myopia prevention and control (e.g., surgery, atropine eye drops, orthokeratology, etc.) (41). Therefore, doctors and eye health practitioners should promote this rule. For example, doctors can educate myopic children and their parents about this method of reducing visual fatigue during outpatient visits, and they can also highlight this rule when producing science popularization works. Then, for 0–17 age group, it is reassuring to see that they have the best eye care habits among all groups. Nonetheless, they still obsess a high prevalence of myopia. This may be related to an increased academic pressure as mentioned above, which will then lead to a series of harmful behaviors including sedentary, increased duration of sustained near eye use and decrease in outdoor activities (45, 46). Therefore, the government and schools need to act together to reduce the excessive academic pressure on young people, and to increase the time and extent of outdoor sports for children in moderation through a rational program of activities. For 18–25 and 26–35 age groups, there is a need for them to know and cultivate the healthy eye care habit, they examine their eyes irregularly and less comprehensively. Considering that for most of these people, they can benefit from medical examinations organized by their school or workplace, although these are often very simple. Thus, these people need to do extra and more comprehensive eye examinations in hospital. Interestingly, for people whose age is more than 35, there is an increase prevalence of hyperopia (20/109) than in younger group (8/284). It is known to all that different from myopia, hyperopia is a situation mostly because of inheritance when excluding the hyperopia secondary to other ophthalmic diseases. Therefore, there may exist presbyopia in these groups, pointing out that these people need to not only pay attention to their myopia, but also to their potential presbyopia.

Studies has demonstrated that spreading of scientific knowledge to the general public has a positive impact on patients' understanding of their diseases and their adherence to treatment (39). Hence, promoting public awareness and education about myopia is crucial for reducing the rate of myopia. A previous study has indicated that the topics of myopia control and myopia management were the most commonly searched for by the public. However, the specific problems related to different age groups have not been specified (47). The differences in concerns regarding myopia among different age groups highlight the significance of customized myopia education. Individuals aged 17 and below, namely children and adolescents, exhibited a greater emphasis on fundamental concerns linked to myopia. This could be attributed to their limited understanding of the subject and their need for basic knowledge regarding myopia. The age range of 18–25 demonstrated a keen interest in myopia management, highlighting a significant inclination toward acquiring knowledge to effectively manage myopia. The age group consisting of individuals between 36 and 45 years old had a higher level of concern regarding rumors pertaining to myopia. This indicates the necessity of prioritizing efforts to provide clear and accurate information regarding myopia-related rumors. The results highlighted the notable variations in concerns related to myopia among different age groups, emphasizing the need for personalized myopia education to offer pertinent knowledge and reduce anxiety about myopia across all age groups. This will contribute to more effective prevention and management of myopia. According to these results, it is necessary to achieve precise science popularization. Some studies have already suggested that online applications dedicated to science popularization can be built, and that the information and browsing habits of users collected through big data can be used to accurately deliver the popularization information that users need. For children with limited access to mobile phones, public service activities can be arranged in schools to disseminate myopia-related knowledge of interest to them (48). Though there are some differences among the age groups, it's worth noting that nearly half of the subjects (184/393) show the worry on rumor about the myopia, which nowadays mainly spread on the Internet. Unfortunately, there haven't been any effective tool on the social media, where most of the rumors are published. But after the COVID-19, a number of tools have emerged that use information technology to identify rumors of COVID-19 or trace them back to their source, and although they have not yet been able to be applied on a large scale in social media to validate effectiveness, reassuring results have been obtained in the laboratory (49–51). In the future, tools that can effectively identify myopia-related rumors can be developed by drawing on the experience of these tools through the intersection of biomedical and industrial. Besides, on the platform “TikTok,” there exists a mechanism named “Crowdsourcing,” meaning fighting against rumors by attracting the professionals who are able to make videos to tell the truth. To diminish the wrong information on the social media, the principles of these platforms could imitate that constructed by TikTok.

This study possesses numerous advantages. Our research is the first to consider the frequency and comprehensiveness of eye examinations at different ages as factors associated with the prevalence of myopia. This facilitates a more intuitive representation of the level of awareness regarding eye care across different age groups, and enables the implementation of online educational and offline screening initiatives that specifically target age groups that do not get eye examinations frequently or comprehensively enough. This not only aids in the prevention or management of myopia, but also offers a chance for early identification of other ocular conditions. Furthermore, our study examines the particular areas of attention about the currently prevalent problem of myopia across various age groups, and identifies differences in concerns among them. This offers valuable insights for future science popularization strategies that aim to target specific demographics using big data, with a focus on precision and effectiveness.

However, it is crucial to acknowledge that this study employed convenience sampling, it did not examine the potential influence of gender and other variables, and the distribution of the online questionnaire may have limited the involvement of older age cohorts. Hence, the limited number of participants aged 60 and above might restrict the generalizability of the findings due to the small sample size. Also, this study didn't examine the region of the subjects, so there maybe exist the potential influence of region on myopia. Finally, to illustrate our results, we exhibit some possible causes based on current studies, but these causes didn't been confirmed in this study. Future research should incorporate probabilistic sampling techniques, increase the sample size as well as carry out longitudinal studies or interventions and investigate in different regions to enhance the credibility of these results. Moreover, to make the results more convinced, it is also necessary to artificially have precise science popularization for each age groups and to compare changes in eye health with a control group after a period of intervention. Researchers should also explore the potential causes of the phenomenon showed in this research.

## 5 Conclusions

The text highlights the importance of providing customized education on myopia for different age groups. It emphasizes the need for innovative approaches and diverse educational content to promote the popularization of myopia science. The government, media, hospitals and schools need to work together to achieve this goal. This, in turn, can contribute to reducing the prevalence of myopia and enhancing the overall vision and health quality of the public. Future studies should utilize probability sampling methods, increase sample sizes, and conduct longitudinal or intervention studies. In addition, studies should also cover subjects from different regions to enhance the credibility of the findings. In order to better validate the findings of this study, it is recommended to further explore the mechanisms of eye health changes by comparing the intervention and control groups.

## Data Availability

The raw data supporting the conclusions of this article will be made available by the authors, without undue reservation.
