# Supplementary material for: A survey of public eye-care behavior and myopia education
Source: Front Public Health. 2025 Feb 18;13:1518956. doi: 10.3389/fpubh.2025.1518956 (PMC11880790; doi:10.3389/fpubh.2025.1518956)
Supplement: Supplementary file 1 [file Supplementary_file_1.docx]

**Survey on the public's visual acuity and awareness of myopia**

1. How old are you？

| □ 0-17 |
| --- |
| □ 18-25 |
| □ 26-35 |
| □ 36-45 |
| □ 46-60 |
| □ above than 60 |

2. Do you have __________?

| □ Myopia |
| --- |
| □ Hyperopia |
| □ Astigmatism |
| □ None |

3. What are your top concerns about myopia?

| □ The etiology of myopia. |
| --- |
| □ Whether myopia is reversible. |
| □ Whether myths of myopia (For example, wearing eyeglasses can worsen myopia, and eye care devices and certain myopia treatment facilities can permanently restore vision.) are true. |
| □ Some notes on methods about myopia prevention and management. |
| □ Other _________________. |

4. What are your opinions about laser eye surgery for myopia?

| □ It is a cure for myopia, and all people is suitable for this surgery. |
| --- |
| □ It is a cure for myopia, and it has surgical indications. |
| □ It's a method for vision improvement, but doesn't cure myopia. |
| □ After laser surgery, there is still a possibility of vision deterioration. |
| □ Other_________________. |

5. How often do you have eye exams?

| □ Semi-annually. |
| --- |
| □ Annually. |
| □ Irregular. |
| □ Never. |
| □ Other _________________. |

6. What does your eye exams typically include?

| □ Comprehensive examinations (include uncorrected and corrected vision acuity, astigmatism examination, eye axis, and fundus photography). |
| --- |
| □ Less comprehensive eye examinations (less than 2 items). |

7. What are your eye habits?

| □ Following the '20-20-20' eye care rule. |
| --- |
| □ Not taking special care of eyes, but relaxing eyes when feeling visual fatigue or when finishing working or studying. |
| □ Using anti-blue light glasses, steam eye masks, eye drops, eye patches and other methods. |
| □ Other _________________ |

8. How do you think the relationship between myopia and genetics?

| □ Related and significant. |
| --- |
| □ There is a relationship, but whether or not a child is myopia is a combination of genetic and environmental (eye habits, etc.) factors. |
| □ It doesn't matter much, there are many cases around where parents are myopic but children have good eyesight. |
| □ Other _________________. |

9. How do you think vision changes in adults?

| □ It's basically set, no matter how frequent the eye use, vision won't change much. |
| --- |
| □ If using eyes excessively or staying in a state of visual fatigue for a long time, myopia will still progress. |
| □ If you have myopia, you won't have presbyopia. |
| □ Other _________________. |

10. What symptoms have you experienced as a result of prolonged Internet classes or staring at electronic screens in the office during COVID-19 (Coronavirus Disease 2019)?

| □ Blurred vision. |
| --- |
| □ Dry, watery eyes. |
| □ Sore eyes. |
| □ Double vision. |
| □ Other_________________. |
